# A Comparison of Azacitidine and Decitabine Activities in Acute Myeloid Leukemia Cell Lines

**DOI:** 10.1371/journal.pone.0009001

**Published:** 2010-02-02

**Authors:** Paul W. Hollenbach, Aaron N. Nguyen, Helen Brady, Michelle Williams, Yuhong Ning, Normand Richard, Leslie Krushel, Sharon L. Aukerman, Carla Heise, Kyle J. MacBeth

**Affiliations:** 1 Celgene Corporation, San Francisco, California, United States of America; 2 Department of Biochemistry and Molecular Genetics, University of Colorado Denver School of Medicine, Aurora, Colorado, United States of America; University of Barcelona, Spain

## Abstract

**Background:**

The cytidine nucleoside analogs azacitidine (AZA) and decitabine (DAC) are used for the treatment of patients with myelodysplastic syndromes and acute myeloid leukemia (AML). Few non-clinical studies have directly compared the mechanisms of action of these agents in a head-to-head fashion, and the agents are often viewed as mechanistically similar DNA hypomethylating agents. To better understand the similarities and differences in mechanisms of these drugs, we compared their *in vitro* effects on several end points in human AML cell lines.

**Methodology/Principal Findings:**

Both drugs effected DNA methyltransferase 1 depletion, DNA hypomethylation, and DNA damage induction, with DAC showing equivalent activity at concentrations 2- to 10-fold lower than AZA. At concentrations above 1 µM, AZA had a greater effect than DAC on reducing cell viability. Both drugs increased the sub-G1 fraction and apoptosis markers, with AZA decreasing all cell cycle phases and DAC causing an increase in G2-M. Total protein synthesis was reduced only by AZA, and drug-modulated gene expression profiles were largely non-overlapping.

**Conclusions/Significance:**

These data demonstrate shared mechanisms of action of AZA and DAC on DNA-mediated markers of activity, but distinctly different effects in their actions on cell viability, protein synthesis, cell cycle, and gene expression. The differential effects of AZA may be mediated by RNA incorporation, as the distribution of AZA in nucleic acid of KG-1a cells was 65∶35, RNA∶DNA.

## Introduction

Azacitidine (AZA; Vidaza®, Celgene Corp., Summit, NJ) and decitabine (DAC; Dacogen®, Eisai Inc., Woodcliff Lake, NJ) are structurally related, but distinct, cytidine nucleoside analogs used clinically for the treatment of myelodysplastic syndromes (MDS) andacute myeloid leukemia (AML) [1], [2]. AZA is a ribonucleoside and DAC is a deoxyribonucleoside [3]. Following cellular uptake and sequential phosphorylations, AZA is incorporated into both RNA and DNA [4]–[6]. In contrast, DAC is phosphorylated by different kinases and is incorporated solely into DNA [6]. Once incorporated into DNA, AZA and DAC have related mechanisms of action, including depletion of DNA methyltransferases (DNMTs) [6], [7], hypomethylation of DNA [8], [9], and induction of DNA damage [10], [11]. In randomized controlled phase III clinical trials in patients with MDS, overall response rates with AZA and DAC have been similar [12]–[15]; however, overall survival rates have differed. Whereas AZA demonstrated a significantly increased median overall survival in higher-risk MDS patients (by 9.4 months) compared with conventional care regimens [14], DAC did not demonstrate a statistically significant improvement in survival in a similar clinical trial [15].

Mechanisms of action that might explain differences in clinical activities of AZA and DAC have not been clearly defined [16]. The conventional description of AZA and DAC as interchangeable DNA hypomethylating agents overlooks potential additional mechanisms of AZA activity which are mediated via incorporation into newly synthesized RNA, including rRNAs, tRNAs, mRNAs, and miRNAs. It has been shown that RNA incorporation can account for 80–90% of the AZA incorporated into cellular nucleic acid [4]. The functional consequences of AZA incorporation into RNA include alterations in the processing of tRNA and rRNAs, leading to inhibition of protein synthesis [5], [17]–[20]. In two recent publications, direct comparisons of AZA and DAC activities have been made [9], [21]. Data support the distinction of AZA and DAC as non-equivalent agents. In one study, the sensitivities (EC_50_ values) of a panel of human cancer cell lines to AZA and DAC showed no correlation, and an AML cell line selected for resistance to DAC remained sensitive to AZA [21]. In another head-to-head *in vitro* comparison of these agents, AZA and DAC had distinct effects on gene expression profiles in Kasumi-1 AML cells [9].

To increase our understanding of the different mechanisms underlying AZA and DAC activity in AML, we directly compared their *in vitro* effects on several end points in human AML cell lines. Specifically, we compared the dose-response effects of AZA and DAC on cell viability, protein synthesis, DNMT1 protein, DNA damage, DNA methylation, cell cycle, apoptosis, and gene expression. Additionally, we tested the relative incorporation of AZA into the DNA and RNA of KG-1a cells. We show that both drugs modulate markers affected by DNA incorporation; however, the drugs have distinctly different effects on cell viability, protein synthesis, cell cycle, and gene expression.

## Methods

### Cell Culture and Drug Treatments

Human AML cell lines (THP-1 and HL-60) and media (RPMI-1640 and MEM) were purchased from American Type Culture Collection (Manassas, VA). Other human AML cell lines (KG-1a and OCI-AML3) were purchased from DSMZ GmbH (Braunschweig, Germany). Cell lines were grown in their respective vendor-recommended culture media and passaged every 3–5 days. In all experiments, cells were seeded approximately 24 hours before drug treatment at 37°C, 5% CO_2_, and cells were treated daily with serial dilutions of freshly-prepared compounds. AZA was manufactured at Aptuit Inc. (Greenwich, CT) for Celgene, and DAC was purchased from Sigma-Aldrich (St. Louis, MO). The concentrations used in experiments include the maximum concentrations (C_max_) achieved in human plasma at clinically used dosages and schedules of administration. Human plasma C_max_ values are 3-11 µM AZA and 0.3–1.6 µM DAC [22], [23], [24]. Radiolabeled AZA, [^14^C]-AZA, was supplied by Aptuit Inc., with the radiolabel on C-4 and a specific activity of 13.7 mCi/mmol.

### Cell Viability

AML cells were seeded in triplicate at 1×10^4^ cells per well in 96-well plates and incubated overnight at 37°C, 5% CO_2_. Cells were treated daily with serial dilutions (0.02–50 µM) of freshly-prepared AZA or DAC and incubated at 37°C, 5% CO_2_ for 72 hours. Cell viability was assessed 72 hours after the initial drug treatment using the CellTiter-Glo assay (Promega, Madison, WI). Luminescence was measured with a spectrophotometer (Molecular Devices, Sunnyvale, CA) at 1500 msec. EC_50_ values were calculated in Prism version 5.01 (GraphPad Software, Inc., La Jolla, CA) using results from two or three independent experiments.

### Western Analysis

AML cells were seeded in either 10-cm dishes at 2.5×10^6^ cells per dish, or 6-well plates at 5×10^5^ cells per well, and incubated overnight at 37°C, 5% CO_2_. Drug treated AML cells were lysed in RIPA buffer (Millipore, Billerica, MA), containing protease inhibitors (Roche Applied Sciences, Indianapolis, IN) and phosphatase inhibitors (Sigma-Aldrich), on ice for 30–60 minutes and then centrifuged to clear cell debris. Protein concentrations were determined using the BCA protein assay (Thermo Fisher Scientific Inc., Waltham, MA). Proteins were separated on Bis-Tris NuPAGE gels and transferred to nitrocellulose membranes. DNMT1, phospho-H2AX, cleaved-PARP, and α-tubulin were detected using the LI-COR Odyssey (LI-COR Biotechnology, Lincoln, NE) imaging system, following incubation with the appropriate primary and secondary antibodies. The phospho-H2AX (Ser 139) and cleaved-PARP antibodies were from Cell Signaling Technology Inc. (Danvers, MA). The α-tubulin and DNMT1 antibodies were from EMD Chemicals Inc. (Gibbstown, NJ) and Abcam Inc. (Cambridge, MA), respectively. The goat anti-rabbit IRDye 680 and goat anti-mouse IRDye 800CW secondary antibodies were from LI-COR. NuPAGE gels were purchased from Life Technologies Corporation (Carlsbad, CA).

### Flow Cytometry

For determination of cell cycle distribution, AML cell lines were stained with NIM-DAPI reagent (Beckman Coulter, Fullerton, CA). Duplicate samples were stained with AnnexinV-FITC and 7-AAD reagents (Beckman Coulter) for determination of early and late apoptotic populations. Samples were processed according to manufacturer's instructions and analyzed on a Beckman Coulter Cell Lab Quanta MPL flow cytometer.

### DNA Methylation Analysis

Genomic DNA was purified from cells using the DNeasy Blood and Tissue Kit (Qiagen, Valencia, CA) according to the manufacturer's instructions. DNA yield was quantitated on a NanoDrop 8000 spectrophotometer (Thermo Fisher Scientific Inc). DNAs (0.5 µg/sample) were submitted to EpigenDx (Worcester, MA) for LINE-1 methylation analysis. LINE-1 methylation was determined by pyrosequencing of bisulfite-converted DNA. Percent LINE-1 methylation represents the average percentage methylation of four CpG sites in duplicate samples. DNAs were submitted to Expression Analysis Inc. (Durham, NC) for array-based methylation analysis of 1505 CpG loci selected from 807 genes (Illumina GoldenGate Methylation Cancer Panel I), according to the manufacturer's instructions. For inclusion in analysis, samples were required to have ≥80% loci (≥1204 loci) with detection p-values<0.05 and a Spearman correlation coefficient of ≥0.7 between biologic duplicates.

### Gene Expression Analysis

Cells were lysed using TRIzol reagent (Life Technologies Corporation) and total RNA was isolated using miRNeasy (Qiagen). Double-stranded cDNA was synthesized using 200 ng of total RNA. Biotin-labeled cRNA was synthesized using MessageAmp aRNA kit (Ambion, Austin, TX), and 15 µg of cRNA was fragmented and hybridized to each human U133A 2.0 gene chipset (Affymetrix, Santa Clara, CA). The GC-RMA algorithm was used for analysis and all analyses were carried out using GeneSpring 7.3 (Agilent, Santa Clara, CA). Averaged signals from biological duplicate samples were used to determine fold-change (treated versus untreated), with absolute fold change of ≥1.7 defining regulated genes. NextBio was used to identify regulated biogroups (based on the Gene Ontology consortium) from lists of regulated genes.

### Incorporation of Radiolabeled AZA into Nucleic Acid

Incorporation of [^14^C]-AZA into the DNA and RNA of KG-1a cells was determined at Southern Research Institute (Birmingham, AL). KG-1a cells (1×10^5^ cells/mL, 3×T75 flasks, 50 mL/flask) were incubated with 0.3 µM [^14^C]-AZA for 24 hours. Radioactive measurement of the trichloroacetic acid (TCA)-precipitable fraction, representing total nucleic acid (RNA + DNA), was performed as previously described [25], [26]. Alkali-stable, TCA-precipitable radioactivity is a measure of the incorporation of nucleosides into DNA. For its determination, cell lysates were incubated with 2N NaOH overnight at 37°C, prior to neutralization, TCA-precipitation, and measurement of radioactivity. Radioactive measurement of the total TCA-insoluble radioactivity minus the alkali-stable TCA-insoluble activity represents the measure of alkali-labile radioactivity in the total TCA precipitate. The alkali-labile fraction of the total TCA precipitate represents RNA.

### Metabolic Labeling

Cells were treated with AZA or DAC for 24 or 48 hours, replacing the media and adding freshly prepared AZA or DAC after 24 hours. Following drug treatments, cells were incubated with methionine/cysteine-free media for 30 minutes. Twenty μCi of ^35^S-methionine and ^35^S-cysteine were then added to cells for 1 hour. Cells were rinsed with methionine/cysteine-free media and then with PBS, prior to lysis in buffer (Promega) with protease inhibitors (Roche). Cell lysates were precipitated with 20% TCA for 1 hour on ice. The precipitate was filtered through a glass microfiber disc and rinsed extensively with cold 20% TCA, followed by cold ethanol. Radioactivity was measured using a scintillation counter. Radioactive counts were normalized to cell numbers, determined in parallel cultures using the CellTiter-Glo assay (Promega).

## Results

### AML Cell Lines Have Differential Sensitivities to AZA Versus DAC

**Figure 1:**
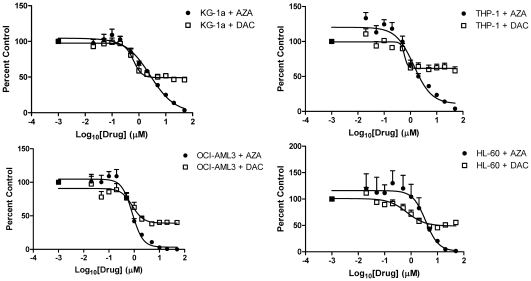
AZA and DAC differentially affect cell viability in AML cell lines. Cell viability of AML cell lines, KG-1a, THP-1, OCI-AML3, and HL-60, was assessed after 72 hours of treatment with AZA (•) or DAC (□) (0–50 µM) using the CellTiter-Glo assay. Standard deviation was determined from 2 or 3 independent experiments, including triplicate wells per experiment. AML  =  acute myeloid leukemia; AZA  =  azacitidine; DAC  =  decitabine.

Four human AML cell lines were assessed for their sensitivity to daily treatment with AZA or DAC in 72 hour cell viability assays (**Figure 1**, **Figure S1**). Dose-response curves and EC_50_ values were established for each drug (**Table 1**). All AML cell lines were sensitive to both drugs, with reduced cell viability observed at concentrations ≥1 µM; however, the maximal amounts of viability reduction with AZA and DAC differed. At high drug concentrations (>1 µM) AZA was consistently more potent than DAC, reducing cell viability to 0–20% at concentrations above 5 µM. DAC, in contrast, did not reduce cell viability below 40% at any concentration up to 50 µM.

**Table 1:** AZA and DAC potencies on acute myeloid leukemia cell viability.

Cell line	AZA EC_50_ ± SEM (µM)	DAC EC_50_ ± SEM (µM)
KG-1a	2.3±0.6	0.4±0.0
THP-1	1.0±0.1	0.5±0.2
OCI-AML3	0.8±0.1	0.7±0.4
HL-60	3.5±0.4	0.8±0.3

EC_50_ values were determined from dose response curves for AZA- and DAC-treated cell lines using Graphpad Prism software.

AZA  =  azacitidine; DAC  =  decitabine; SEM  =  standard error of the mean.

The differential activity of AZA compared with DAC may be explained by differences in the targeted cell sub-populations of asynchronously growing cell cultures. The half-lives of AZA and DAC in cell culture are short (∼8–12 hours), necessitating daily treatment to ensure continued exposure (data not shown). AZA may have activity in cells during all phases of the cell cycle via RNA incorporation, whereas DAC incorporation into DNA is restricted to the S-phase and may limit the number of affected cells at any given treatment time. To test the hypothesis that additional treatments with DAC could further reduce cell viability, a dose-response evaluation was performed in KG-1a cells at 2, 3, 4, 5 and 6 days, with daily DAC addition (**Figure S2**). Cell viability was further reduced at each later time point, with a reduction to ∼15% cell viability by 6 days. These data demonstrate that to achieve a similar reduction in cell viability with DAC versus AZA, cells must be more extensively treated.

### AZA Is Incorporated into Both RNA and DNA in KG-1a Cells

**Figure 2:**
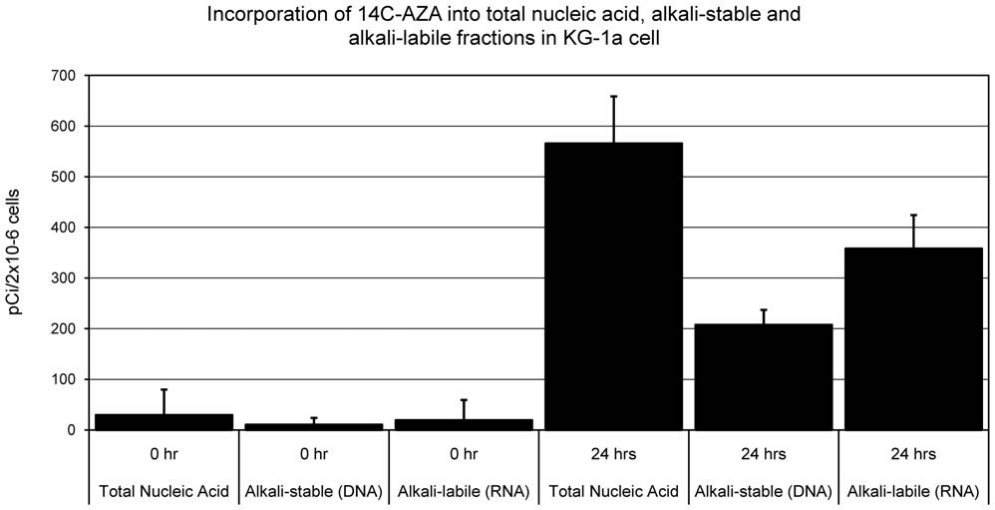
AZA incorporates into RNA and DNA of KG-1a cells. KG-1a cells were treated with 0.3 µM radiolabeled AZA ([^14^C]-AZA) for 24 hours. The amount of AZA incorporated into total nucleic acid, DNA, and RNA was quantified as described previously. Standard error of the mean was determined from 3 independent experiments, including triplicate wells per experiment. AZA  =  azacitidine.

A previous study in a mouse leukemia cell line demonstrated that AZA incorporated into RNA and DNA at a ratio of approximately 85∶15, respectively [4]. To determine the relative distribution of AZA into RNA and DNA in a human AML cell line, we measured incorporation of radiolabeled AZA ([^14^C]-AZA) into total nucleic acid, RNA (alkali-labile nucleic acid) and DNA (alkali-stable nucleic acid) of KG-1a cells (**Figure 2**). [^14^C]-AZA was incorporated into both RNA and DNA of KG-1a cells in a time-dependent manner (data not shown). After a 24 hour incubation with 0.3 µM [^14^C]-AZA, the radioactivity incorporated into the nucleic acid was distributed at a ratio of 65∶35, RNA∶DNA. [^14^C]-DAC, with an appropriately labeled carbon, was not available for direct comparison. These data confirmed the expectation that AZA incorporates into both RNA and DNA in a human AML cell line, with predominant incorporation into RNA compared with DNA.

### AZA and DAC Have Different Effects on Protein Synthesis

**Figure 3:**
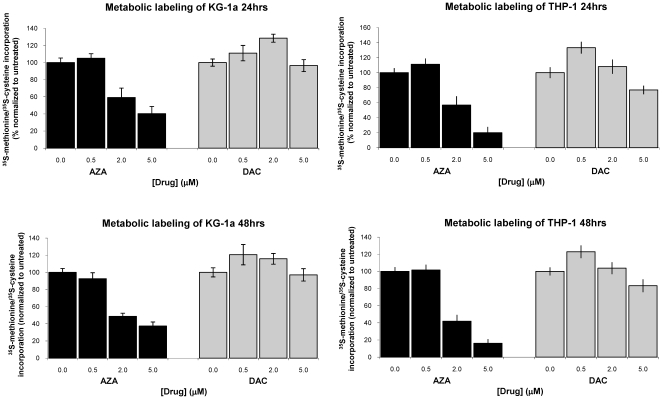
AZA inhibits protein synthesis in KG-1a and THP-1 cells. Cells were treated daily with AZA or DAC (0–5 µM) for 24 or 48 hours prior to metabolic labeling with ^35^S-methionine and ^35^S-cysteine. Protein synthesis was quantified as described previously. AZA  =  azacitidine; DAC  =  decitabine.

Protein synthesis inhibition via RNA incorporation of AZA has been described as a mechanism of AZA activity [5]. The effects of AZA and DAC on protein synthesis were compared by metabolic labeling (^35^S-methionine and ^35^S-cysteine) of KG-1a and THP-1 cells after 24 and 48 hours of daily drug treatment (**Figure 3**). AZA (2 µM) treatment significantly reduced protein synthesis in both cell lines, inhibiting protein synthesis at 48 hours by 51% and 58% in KG-1a and THP-1 cells, respectively. DAC did not reduce protein synthesis in either cell line at 2 µM. Significant inhibition of protein synthesis with AZA (2 µM), but not DAC, was also seen at 24 hours, with synthesis reduced by 41% and 43% in KG-1a and THP-1 cells, respectively. Notably, the AZA concentrations that affected protein synthesis (2–5 µM) were also concentrations at which greater effects on cell viability were observed for AZA versus DAC.

### AZA and DAC Cause Depletion of DNMT1 Protein and DNA Hypomethylation in KG-1a and THP-1 Cells

**Figure 4:**
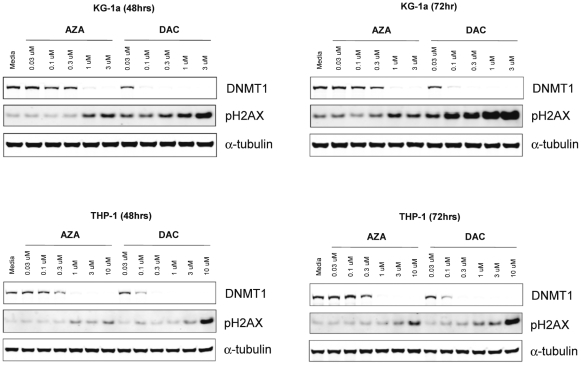
AZA and DAC cause DNMT1 depletion and induction of DNA damage in KG-1a and THP-1 cells. Cells were treated daily with AZA or DAC (0–3 µM in KG-1a; 0–10 µM in THP-1) for 48 and 72 hours. Protein lysates were analyzed by Western analysis for DNMT1 and phospho-H2AX (Ser 139) proteins. α-Tubulin is shown as a protein loading control. AZA  =  azacitidine; DAC  =  decitabine; DNMT  =  DNA methyltransferase.

DNA-mediated effects of AZA and DAC were evaluated, using DNMT1 protein depletion and DNA hypomethylation as markers of drug incorporation into the DNA of KG-1a and THP-1 cell lines. In each cell line, DNMT1 protein was reduced by both AZA and DAC in a dose-dependent manner (**Figure 4**). Complete DNMT1 depletion, as measured by Western analysis, was achieved with lower concentrations of DAC (0.1–0.3 µM), in comparison to AZA (1 µM). DNMT1 protein depletion occurred at clinically relevant drug concentrations. Similar effects on DNMT1 depletion were observed at 48 and 72 hour time points.

**Figure 5:**
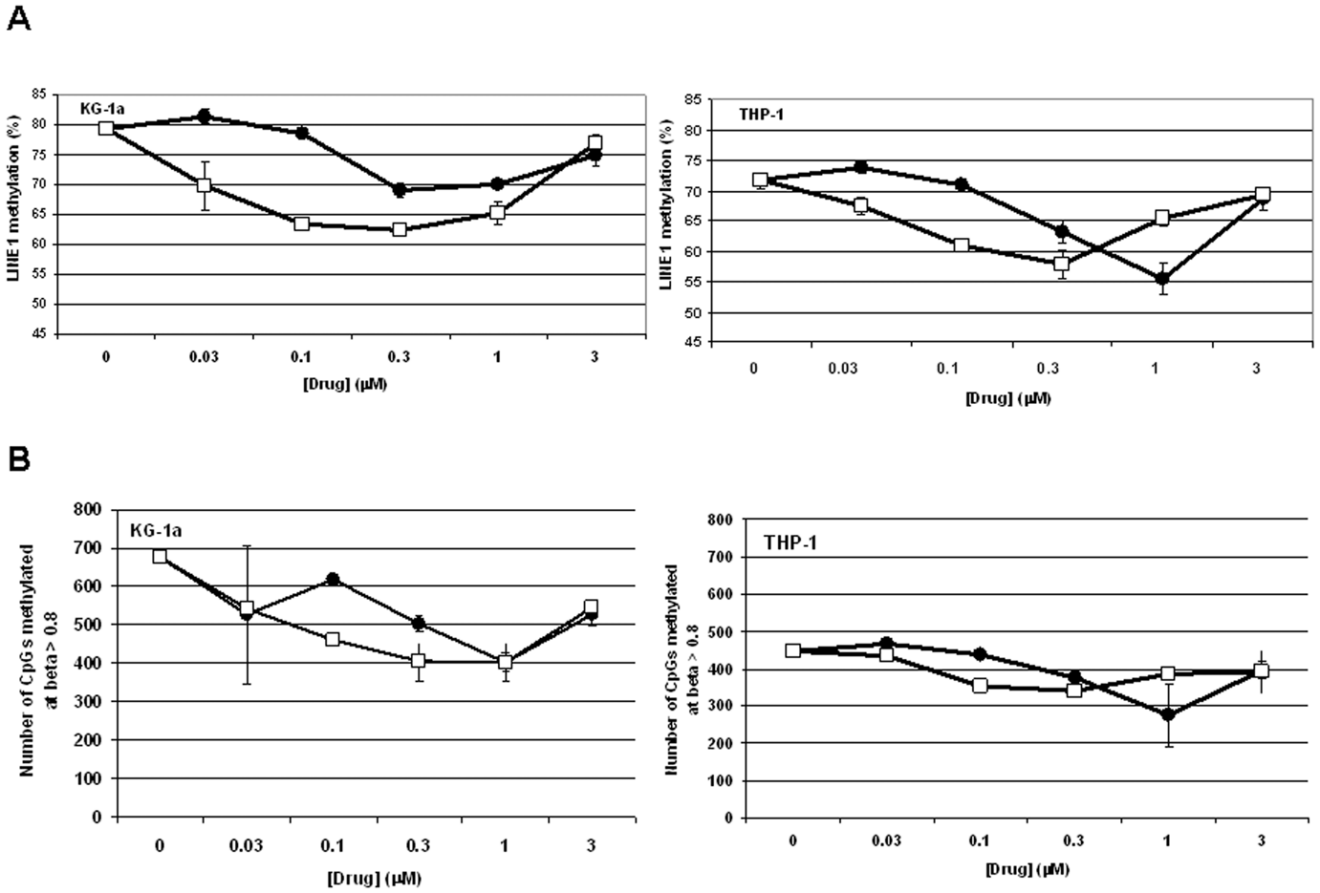
AZA and DAC reduce DNA methylation in KG-1a and THP-1 cells. Cells were treated daily with AZA (•) or DAC (□) (0–3 µM) for 48 hours. DNA methylation was measured using (**A**) pyrosequencing of LINE-1 DNA elements in bisulfite-converted DNA and (**B**) Illumina GoldenGate platform. AZA  =  azacitidine; DAC  =  decitabine.

DNA methylation was measured in drug-treated (48 hour) cells using pyrosequencing of LINE-1 DNA elements in bisulfite-converted DNA. DNA methylation of LINE-1 repeat elements serves as a surrogate measure of global DNA methylation. A decrease in LINE-1 DNA methylation was observed at AZA concentrations of 0.3–1 µM and DAC concentrations of 0.03–1 µM, with maximal hypomethylation observed at concentrations of approximately 1 µM AZA and 0.3 µM DAC in both cell lines (**Figure 5A**). The doses inducing maximal hypomethylation paralleled the doses that maximally depleted DNMT1 protein. In addition to evaluating changes in the LINE-1 DNA methylation, we also evaluated DNA methylation changes at 1505 gene-specific CpG loci using the Illumina GoldenGate DNA methylation platform (**Table S1**). Methylation changes were summarized by plotting the number of highly methylated loci, defined as loci with beta scores >0.8, versus drug concentration. Similar to findings with LINE-1 DNA methylation, the GoldenGate assay showed the greatest reduction in highly methylated CpG loci at concentrations of 1 µM AZA and 0.3 µM DAC in both cell lines (**Figure 5B**). Similar changes in DNA methylation were observed with 72 hour drug treatments, using both DNA methylation assays (data not shown).

### AZA and DAC Induce DNA Damage in KG-1a and THP-1 Cells

Induction of DNA damage by AZA and DAC was measured using phospho-H2AX (Ser 139) as a marker of double stranded DNA breaks. In drug-treated KG-1a and THP-1 cells, dose- and time-dependent induction of phospho-H2AX was observed with both AZA and DAC (Figure 4). In KG-1a cells, induction of phospho-H2AX above basal levels was observed at AZA concentrations ≥1 µM at 48 and 72 hour time points. DAC, in contrast, caused a significantly greater increase in phospho-H2AX at lower drug concentrations (≥0.03 µM). Notably, DNA damage was induced at clinically-relevant drug concentrations for both drugs. Similar results were seen in the THP-1 cell line, with DAC having greater potency than AZA at inducing DNA damage; however, higher AZA (3–10 µM) and DAC (1–3 µM) concentrations were required to induce significant DNA damage in THP-1 cells, in comparison to KG-1a cells.

### AZA and DAC Have Differential Effects on Cell Cycle in KG-1a Cells

**Figure 6:**
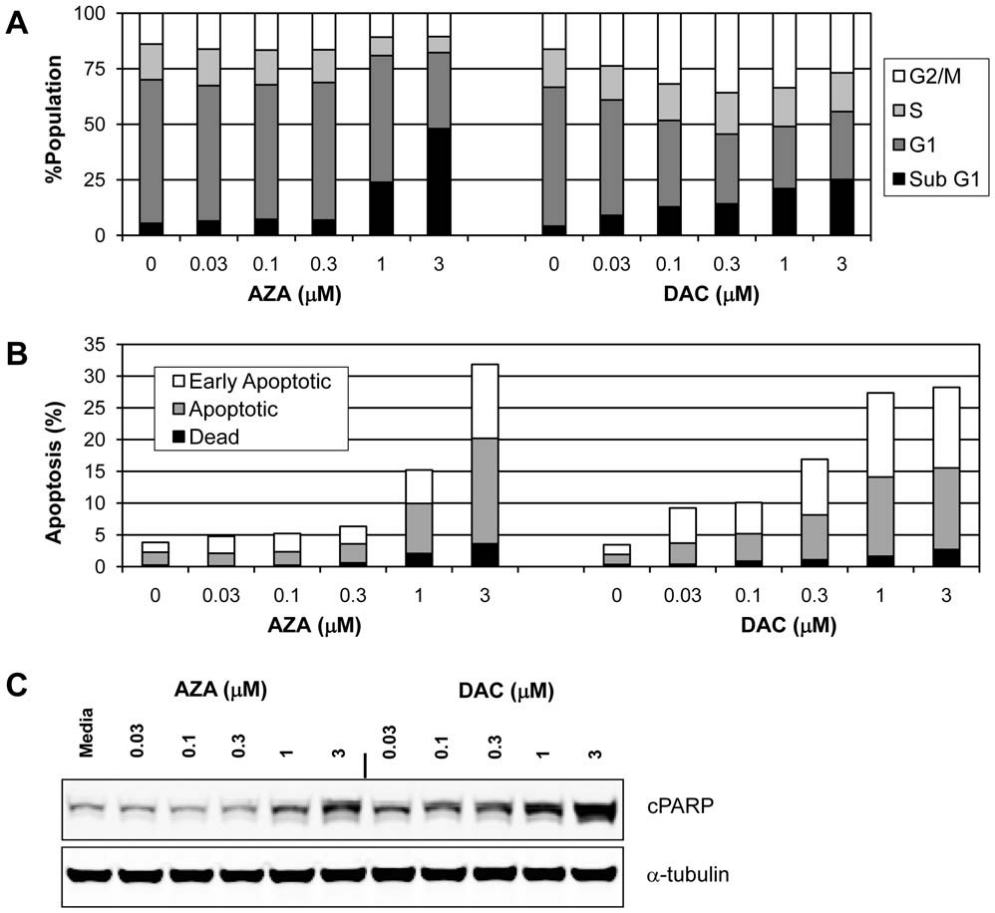
Effects of AZA and DAC on cell cycle and apoptosis in KG-1a cells. KG-1a cells were treated daily with AZA or DAC (0–3 µM) for 48 hours. (**A**) Cell cycle effects of AZA and DAC. Cells were stained with NIM-DAPI and quantification by flow cytometry for percentage of cells in sub-G1, G0/G1, S, and G2-M phases (normalized to 100%). (**B**) AZA and DAC induce apoptosis in KG-1a cells. Apoptosis was detected with flow cytometry by positive staining for Annexin V (early apoptosis) and 7-AAD (late apoptosis). (**C**) Protein lysates were analyzed by Western analysis for detection of PARP cleavage. α-Tubulin is shown as a protein loading control. AZA  =  azacitidine; DAC  =  decitabine.

To better understand the differential effects observed with AZA and DAC in cell viability assays, we analyzed drug-treated (48 hours) KG-1a cells for dose-dependent changes in cell cycle by flow cytometry of NIM-DAPI-stained cells (**Figure 6A**). AZA concentrations below 1 µM had no significant effect on cell cycle, whereas AZA concentrations of 1 µM or greater caused an increase in the sub-G1 fraction of cells and a concomitant decrease in all other phases of the cell cycle. DAC dose-dependently increased the sub-G1 phase; however, in contrast to AZA, DAC also increased the G2-M phase, with a concomitant decrease in the G0/G1 phase. Maximal increase in the G2-M fraction of cells occurred with 0.3 µM DAC. Similar results were observed at a 72 hour time point (data not shown).

### AZA and DAC Increase Markers of Apoptosis in KG-1a Cells

The observation that AZA and DAC treatment cause an increase in the sub-G1 phase of the KG-1a cell cycle prompted us to explore drug-induced effects on markers of apoptosis. Dose-dependent effects of AZA and DAC on markers of apoptosis were evaluated by flow cytometry of KG-1a cells treated for 48 hours and stained with AnnexinV-FITC and 7-AAD to detect early and late apoptotic events (**Figure 6B**). PARP cleavage was also evaluated by Western analysis (**Figure 6C**). An increase in the percentage of KG-1a cells undergoing apoptosis was detected by both flow and Western analyses with AZA (≥1 µM) and DAC (≥0.03 µM). Similar results were observed at the 72 hour time point (data not shown). In both analyses, DAC was more potent than AZA at increasing markers of apoptosis. The greater cell kill observed with AZA versus DAC in viability assays (Figure 1), despite less effect on markers of apoptosis, suggests that mechanisms other than apoptosis are contributing to AZA-mediated cell death.

### AZA and DAC Regulate Different Genes in KG-1a Cells

**Figure 7:**
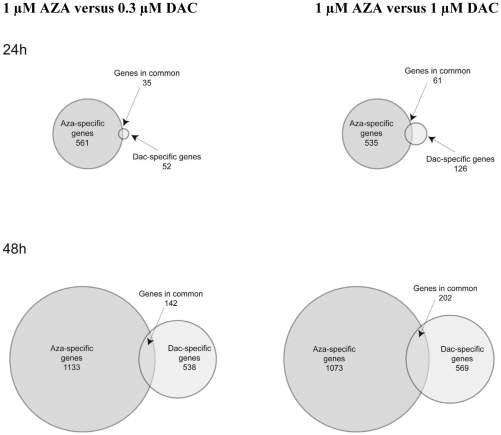
AZA and DAC regulate different genes in KG-1a cells. Venn diagrams reveal the number of genes that are distinctly and commonly regulated by daily treatment with AZA (1 µM) or DAC (0.3 µM or 1 µM) in KG-1a cells at 24 and 48 hours. AZA  =  azacitidine; DAC  =  decitabine.

To further explore similarities and differences in the mechanisms of action of AZA compared with DAC, the molecular pathways regulated by each drug were explored using gene-expression profiling of KG-1a cells treated with a dose range (0.3–3 µM) of each drug for 24 and 48 hours. Genes with an absolute fold change of ≥1.7 following drug treatment were defined as regulated genes. As shown in **Table 2**, AZA regulated few genes at 0.3 µM; however, higher concentrations (1–3 µM) significantly increased the number of genes regulated. DAC regulated more genes than AZA only at 0.3 µM for 48 hours. Generally, AZA (1–3 µM) regulated a greater number of genes compared with DAC (1–3 µM). Gene expression values are provided in **Table S2**. Venn analysis of the genes modulated by each drug revealed that the majority of genes regulated by AZA and DAC are drug-specific (**Figure 7**). Equimolar concentrations (1 µM), as well as concentrations approximating equipotency on DNA hypomethylation (1 µM AZA versus 0.3 µM DAC), were compared. When comparing 1 µM concentrations at 24 hours, the number of uniquely regulated genes represented 90% and 67% of the total number of genes regulated by AZA and DAC, respectively.

**Table 2:** Number of genes regulated by AZA and DAC in KG-1a cells.

Time (hours)	Dose (µM)	AZA-regulated genes	DAC-regulated genes
24	0.3	66	87
24	1	596	187
24	3	1340	331
48	0.3	145	680
48	1	1275	771
48	3	1513	1145

Cells were treated daily with AZA or DAC (0.3−3 µM) for 24 and 48 hours, and RNA was isolated for evaluation of gene expression using Affymetrix human U133A 2.0 gene chipset. The table shows the number of genes regulated by AZA and DAC at different drug concentrations. Duplicate samples of each were averaged and compared with untreated samples. A fold change of ≥1.7 in gene expression was considered as regulated.

AZA  =  azacitidine; DAC  =  decitabine.

Lists of the drug-regulated genes were analyzed using NextBio in order to identify the affected gene ontology biogroups. **Table 3** lists the biogroups that were most significantly regulated by AZA and DAC in KG-1a cells treated for 24 and 48 hours. The biogroups most significantly regulated at each time point were different for AZA and DAC. AZA (1 µM) most significantly regulated biogroups representing metabolic processes, aminoacyl-tRNA ligase activity and mitochondrion at 24 hours, as well as mitosis, cell cycle, and cell division at 48 hours. In contrast, DAC (1 µM) significantly upregulated the cell differentiation biogroup at both 24 and 48 hours. The biogroup of genes representing aminoacyl-tRNA ligase activity was significantly regulated by both AZA and DAC; however, AZA upregulated this biogroup at 24 hours, while DAC downregulated this biogroup at 48 hours.

**Table 3:** Gene biogroups significantly regulated by AZA or DAC in KG-1a cells.

GO category	1 µM AZA, 24 hours*P*-value (direction)	1 µM DAC, 24 hours*P*-value (direction)
Sterol metabolic process	1.0E-15 (down)	
Ligase activity, forming aminoacyl-tRNA and related compounds	3.8E-12 (up)	
Lipid metabolic process	4.1E-11 (down)	
Mitochondrion	3.3E-10 (down)	0.0023 (down)
Cell differentiation	0.0014 (up)	2.1E-9 (up)
Co-factor binding	1.9E-8 (up)	4.3E-7 (down)

GO category	1 µM AZA, 48 hours*P*-value (direction)	1 µM DAC, 48 hours*P*-value (direction)
Mitosis	3.0E-53 (down)	
Cell cycle	9.1E-46 (down)	0.0088 (down)
Cell division	4.9E-44 (down)	
Chromosome	2.6E-26 (down)	
Response to DNA damage stimulus	8.2E-22 (down)	0.0002 (down)
Ligase activity, forming aminoacyl-tRNA and related compounds		2.4E-17 (down)
Cytoskeleton	2.3E-12 (down)	0.0006 (up)
Cell differentiation	0.0024 (up)	4.4E-12 (up)

Biogroups of genes regulated ≥1.7-fold by daily treatment with AZA (1 µM) or DAC (1 µM) in KG-1a cells at 24 and 48 hours. Directionality indicates the predominant direction of gene regulation within each biogroup. P-values of low significance are included for biogroups regulated by both drugs, but highly significant for only one drug.

AZA  =  azacitidine; DAC  =  decitabine.

## Discussion

In human AML cell lines we compared dose-dependent responses to AZA and DAC on cell viability, protein synthesis, DNMT1 depletion, hypomethylation of DNA, induction of DNA damage, cell cycle, induction of apoptosis, and gene expression. Both AZA and DAC regulated molecular end points related to drug incorporation into DNA, including DNMT1 depletion, DNA hypomethylation, and induction of the DNA damage marker phospho-H2AX. DAC affected these DNA-mediated markers at concentrations 2- to 10-fold lower than those of AZA, likely due to greater incorporation of DAC into DNA [4], [27]. Previous direct comparisons of the DNA hypomethylating activities of AZA and DAC have also shown that DAC is more potent in this regard [9], [28]. The experiment in KG-1a cells evaluating AZA incorporation into RNA and DNA showed a distribution of 65% and 35%, respectively. If the rates of cellular uptake and nucleic acid incorporation for AZA and DAC are similar, a 3-fold decrease in potency on DNA-mediated markers would be expected when comparing equimolar amounts of AZA versus DAC. Differences in clinical dosing and scheduling may influence the extent of DNA-mediated activities of these drugs in patients.

Differences between the mechanisms of action of AZA and DAC were observed in their activities on cell viability, protein synthesis, cell cycle, and gene expression. Consistent differences in the dose-response curves of AZA compared with DAC on cell viability were observed in four human AML cell lines, with AZA having a greater effect than DAC at reducing cell viability at drug concentrations above 1 µM. Clinically achievable plasma concentrations are 3–11 µM AZA and 0.3–1.6 µM DAC [22], [23], [24]. It is important to note that AZA and DAC both caused depletion of DNMT1 protein and DNA hypomethylation, within the timeframe of the cell viability assessment; therefore, the differential effects on cell viability cannot be accounted for solely by epigenetic mechanisms. The greater potency of DAC versus AZA, based on calculated EC_50_ values, does not take into account the plateau effect on cell viability observed with DAC, in which increasing drug concentrations above 1 µM did not lead to a further reduction in cell viability below 40% after 3 days. This plateau effect with DAC likely reflects the fact that DAC activity is specific to DNA incorporation in the S-phase of the cell cycle [29], and treating cells for additional days could further reduce cell viability. Although AZA incorporation into DNA would similarly be S-phase restricted, AZA incorporation into RNA should occur in all phases of the cell cycle. In fact, previous studies showed that AZA (2–8 µM) induction of apoptosis in the human AML cell line HL-60 was preferential to G1-phase cells and occurred in a short timeframe (4–8 hours), implicating an RNA mechanism [30], [31].

AZA inhibited protein synthesis at 24 hours post-treatment, an effect occurring within the doubling time of these cells. Earlier time points were not evaluated. DAC, in contrast, did not inhibit protein synthesis. The inhibition of protein synthesis by AZA was observed at concentrations that reduced cell viability below that of DAC, suggesting that the anti-leukemic activity of AZA observed at drug concentrations >1 µM may be explained by mechanisms in addition to, or other than, DNA-mediated mechanisms. In multiple myeloma cell lines, AZA reduced IL6-Rα protein levels within 2 hours, and to an equal extent as cycloheximide, consistent with a mechanism involving protein synthesis inhibition [32].

Differences in cell cycle regulation with AZA and DAC were also observed in KG-1a cells. Although both drugs increased the sub-G1 fraction of cells, DAC caused a concomitant increase in G2-M and decrease in G0/G1 phases of the cell cycle, whereas AZA decreased all phases of the cell cycle. The increase in G2-M phase with DAC in KG-1a cells is consistent with previous observations in other hematopoietic tumor cell lines [33], [34]. Both drugs increased markers of apoptosis, including Annexin V staining and cleaved-PARP. Increased apoptosis has also been observed with AZA and DAC treatment of other leukemic cell lines [11], [30], [31], [33], [35]–[37]. The lesser effect of AZA compared with DAC on markers of apoptosis, despite greater cell killing with AZA, suggests that mechanisms other than apoptosis are contributing to AZA-mediated programmed cell death.

Finally, microarray analysis of gene expression in KG-1a cells revealed differences between AZA and DAC. At low concentrations (<0.3 µM), very few genes were regulated at the 24 and 48 hour time points by either drug (data not shown). In contrast, at high concentrations (≥1 µM) over 1000 genes could be regulated (Table 2), and AZA regulated a greater number of genes compared with DAC. The concentrations of AZA that led to significant gene modulation were also the concentrations that affected cell viability. When comparing the lists of genes that were regulated by AZA (1 µM) and DAC (0.3 and 1 µM), a minority of genes were commonly regulated. It should be noted that 5–25% of the genes identified as “commonly regulated” in the overlapping Venn diagram were regulated by AZA and DAC in opposite directions, further suggesting mechanistic differences between these drugs. Our findings are consistent with a recently published gene expression comparison of AZA and DAC in the AML cell line Kasumi-1, although low concentrations of each drug (0.5 µM AZA and 0.05 µM DAC) were used in that study [9]. Pathway analysis of the regulated genes provided intriguing insights into the cellular actions of these drugs. AZA significantly downregulated genes involving cell cycle, cell division and mitosis, whereas DAC significantly upregulated genes involved in cell differentiation (Table 3).

These data illustrate similarities and differences in the mechanisms of action of AZA and DAC. Historically, these drugs have been viewed as mechanistically similar DNA hypomethylating agents, and both have been described as having dose-dependent, dual mechanisms of action [1], [2]. For DAC, the “dual mechanism” has referred to inhibition of cell proliferation at high doses and a DNA hypomethylation-mediated effect on gene re-expression at low doses, affecting processes of cell differentiation, tumor suppression, and stimulation of immune mechanisms [2]. For AZA, the “dual mechanism” has referred to cytotoxicity at high doses, via RNA and DNA incorporation, and DNA hypomethylation at lower doses [1]. Certainly both drugs have dose-dependent effects; however, previously described “dual mechanisms” of these drugs should not be interpreted as shared. We show that both drugs modulated markers of azanucleoside incorporation into DNA (DNMT1 depletion, DNA damage induction, and DNA hypomethylation); however, DAC demonstrated a greater effect on these markers. Also, although AZA and DAC increased the sub-G1 fraction of cells and markers of apoptosis, AZA demonstrated a greater effect on reducing cell viability and decreasing protein synthesis. It is clear that the anti-leukemic activities of AZA and DAC differ *in vitro*, with DAC acting solely through DNA-mediated mechanisms (epigenetic and/or DNA damage), and AZA acting via mechanisms in addition to, or other than, incorporation into DNA. Translational research will be key to understanding how the mechanistic differences observed between AZA and DAC *in vitro* will be best applied to the clinical utility of these drugs.

## Supporting Information

Figure S1 AZA and DAC differentially affect cell viability in AML cell lines. Cell viability of AML cell lines, KG-1a and THP-1, was assessed after 72 hours of daily treatment with AZA or DAC (0–50 µM), using direct cell counts with trypan blue exclusion or MTS assay. Standard deviation was determined from triplicate wells of a single experiment, except for the KG-1a direct count data, which shows error as the range of duplicate wells.
(0.11 MB PPT)

Figure S2 Extended dosing with DAC further reduces KG-1a cell viability. KG-1a cell viability was assessed at 2, 3, 4, 5 and 6 days, with daily DAC addition, using the CellTiter-Glo assay.
(0.14 MB PPT)

Table S1 DNA methylation (Illumina GoldenGate Methylation Cancer Panel I) Beta values in KG-1a and THP-1 cells at 48 and 72 hours, following daily treatment with vehicle control, AZA and DAC at 0.03, 0.1, 0.3, 1 and 3 µM. AZA  =  azacitidine; DAC  =  decitabine.
(2.35 MB XLS)

Table S2 Gene expression (Affymetrix U133A 2.0 gene chipset) values in KG-1a cells at 24 and 48 hours, following daily treatment with vehicle control, AZA and DAC (0.3, 1 and 3 µM). AZA  =  azacitidine; DAC  =  decitabine.
(22.84 MB XLS)
