# Supplementary material for: A Comparison of Azacitidine and Decitabine Activities in Acute Myeloid Leukemia Cell Lines
Source: PLoS One. 2010 Feb 2;5(2):e9001. doi: 10.1371/journal.pone.0009001 (PMC2814859; doi:10.1371/journal.pone.0009001)
Supplement: Figure S1 — AZA and DAC differentially affect cell viability in AML cell lines. Cell viability of AML cell lines, KG-1a and THP-1, was assessed after 72 hours of daily treatment with AZA or DAC (0–50 µM), using direct cell counts with trypan blue exclusion or MTS assay. Standard deviation was determined from triplicate wells of a single experiment, except for the KG-1a direct count data, which shows error as the range of duplicate wells. (0.11 MB PPT) [file pone.0009001.s001.ppt]

## Slide 1
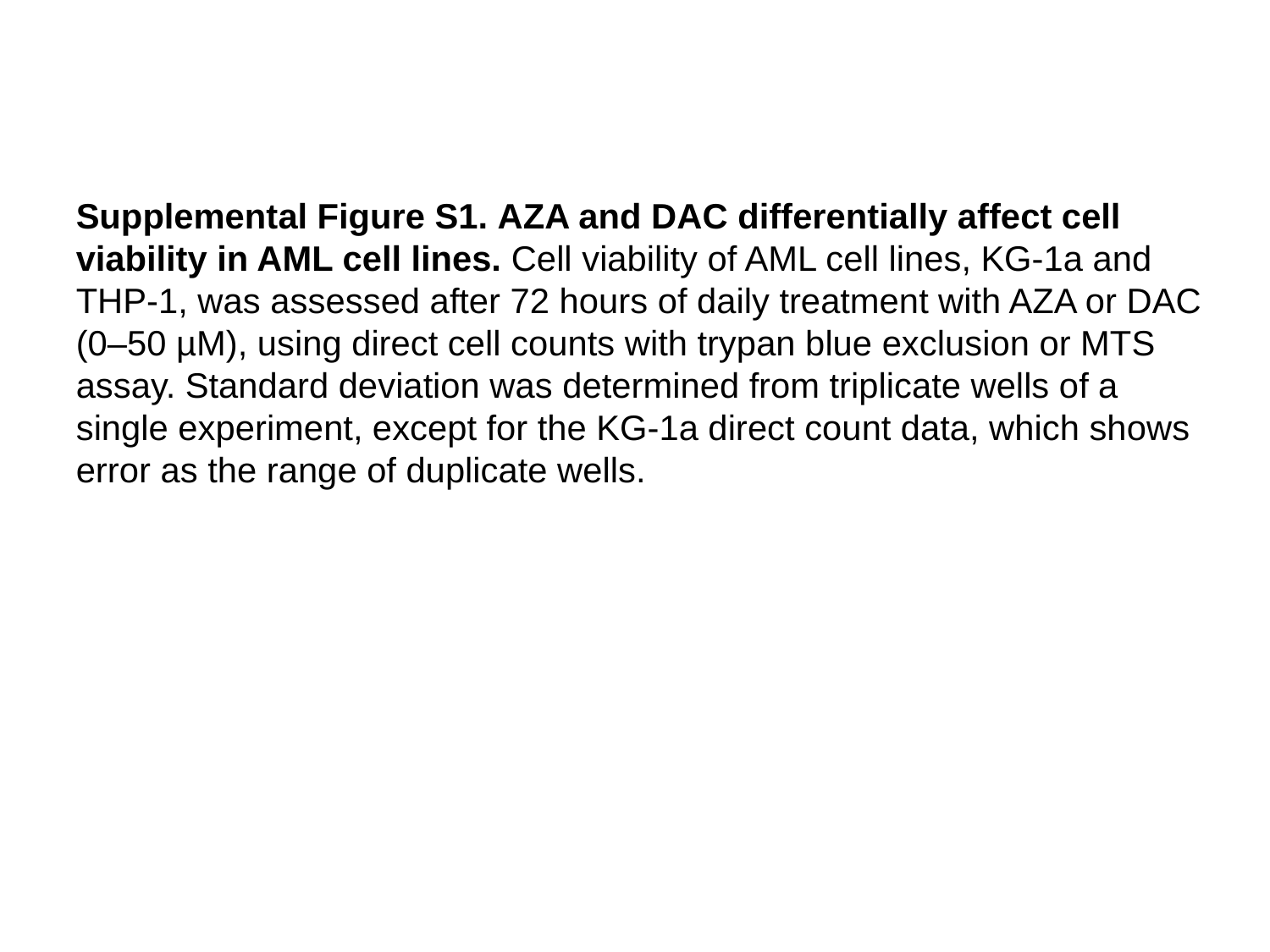

# Supplemental Figure S1. AZA and DAC differentially affect cell viability in AML cell lines. Cell viability of AML cell lines, KG-1a and THP-1, was assessed after 72 hours of daily treatment with AZA or DAC (0–50 µM), using direct cell counts with trypan blue exclusion or MTS assay. Standard deviation was determined from triplicate wells of a single experiment, except for the KG-1a direct count data, which shows error as the range of duplicate wells.

## Slide 2
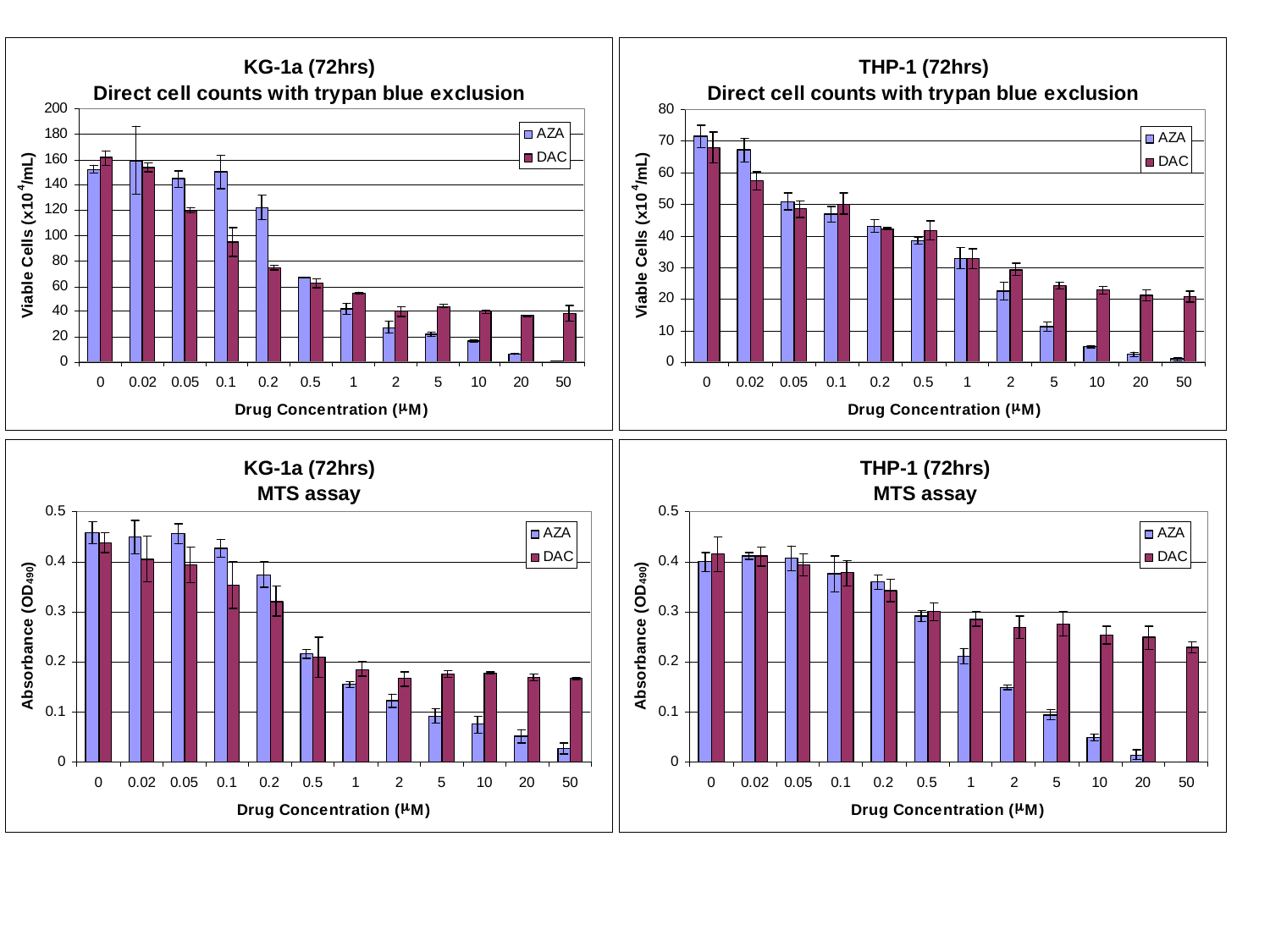

## Slide 3
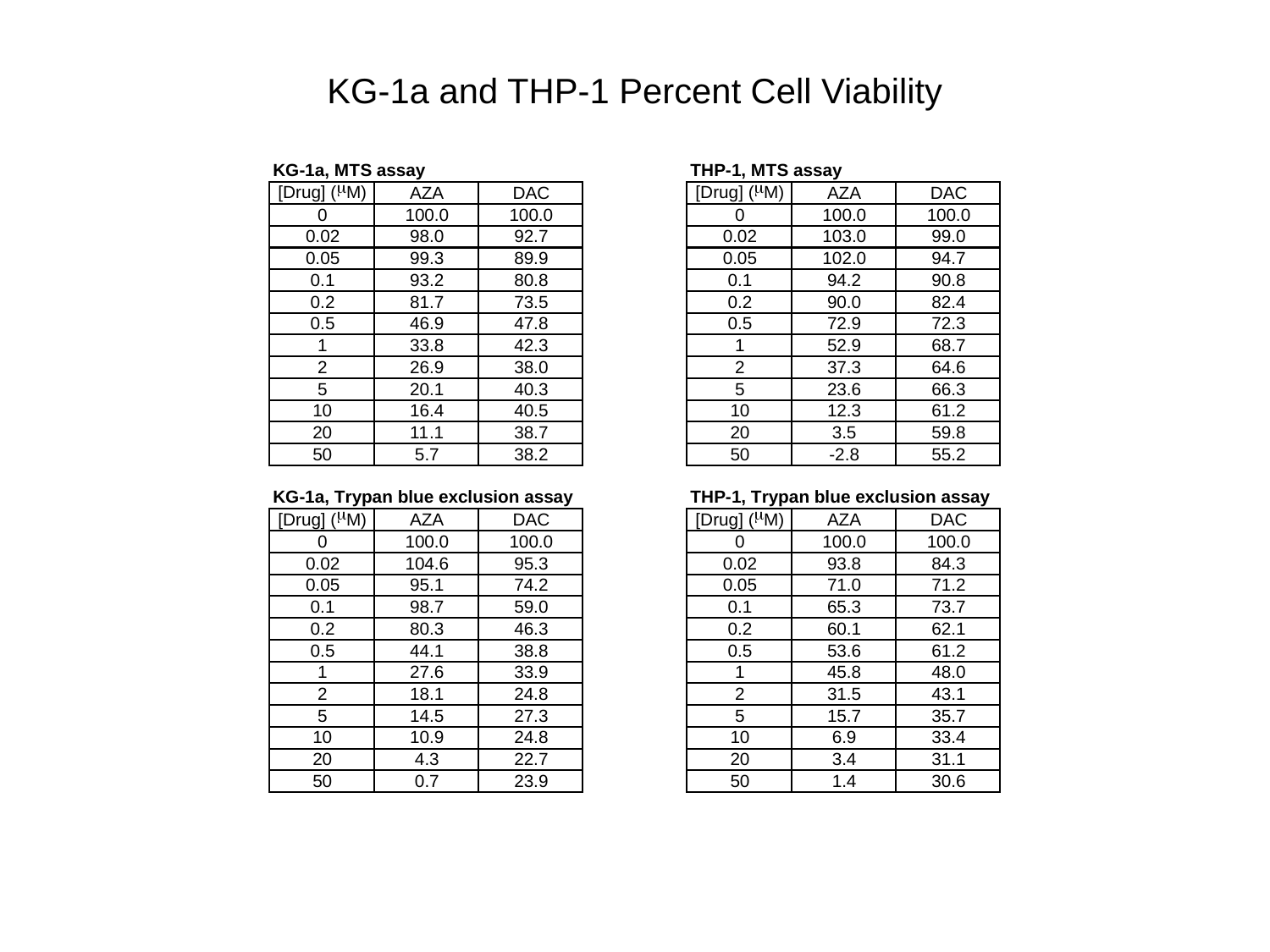

# KG-1a and THP-1 Percent Cell Viability
