# Supplementary material for: A Comparison of Azacitidine and Decitabine Activities in Acute Myeloid Leukemia Cell Lines
Source: PLoS One. 2010 Feb 2;5(2):e9001. doi: 10.1371/journal.pone.0009001 (PMC2814859; doi:10.1371/journal.pone.0009001)
Supplement: Figure S2 — Extended dosing with DAC further reduces KG-1a cell viability. KG-1a cell viability was assessed at 2, 3, 4, 5 and 6 days, with daily DAC addition, using the CellTiter-Glo assay. (0.14 MB PPT) [file pone.0009001.s002.ppt]

## Slide 1
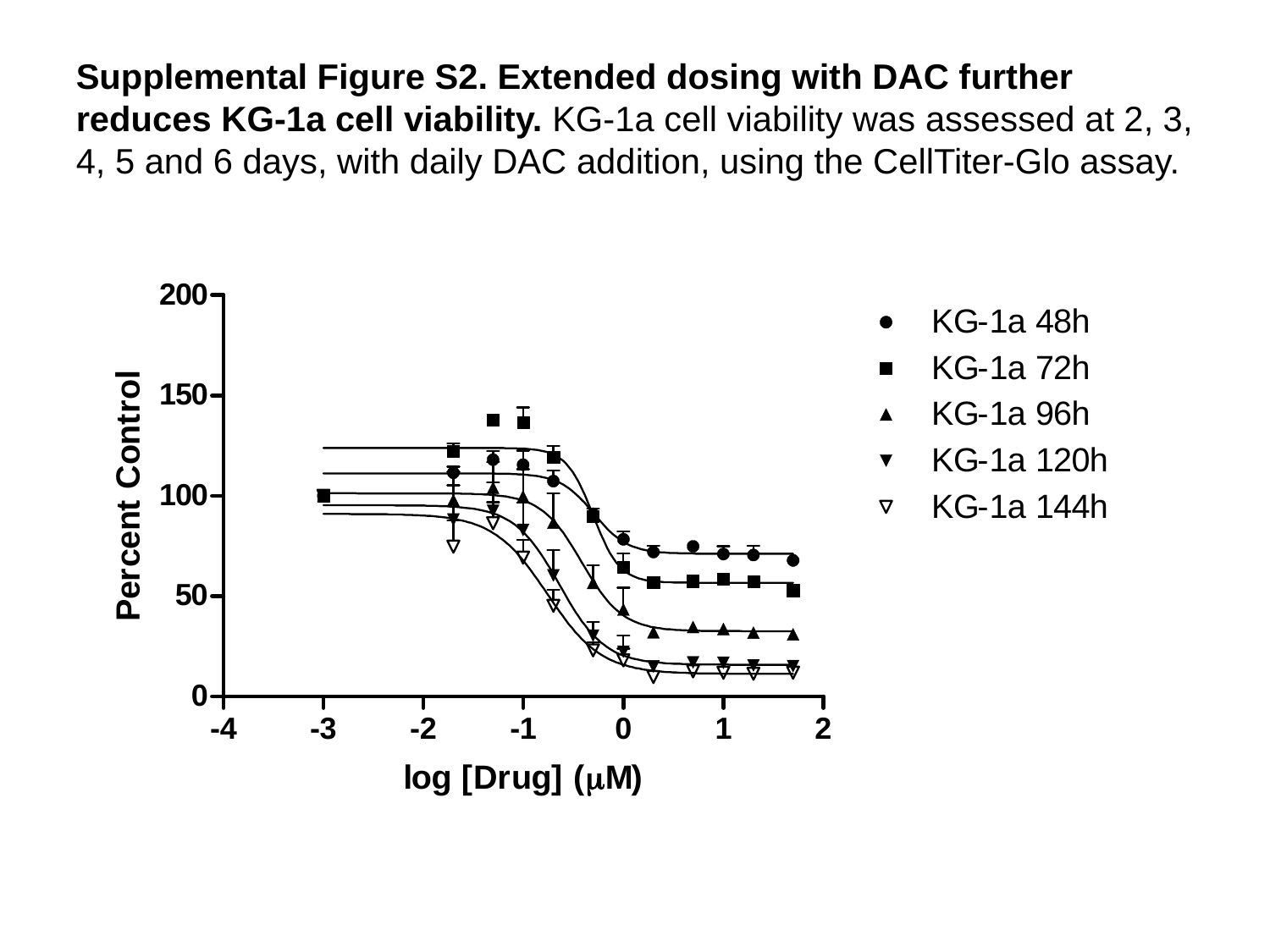

# Supplemental Figure S2. Extended dosing with DAC further reduces KG-1a cell viability. KG-1a cell viability was assessed at 2, 3, 4, 5 and 6 days, with daily DAC addition, using the CellTiter-Glo assay.
